# HyperCys: A Structure- and Sequence-Based Predictor of Hyper-Reactive Druggable Cysteines

**DOI:** 10.3390/ijms24065960

**Published:** 2023-03-22

**Authors:** Mingjie Gao, Stefan Günther

**Affiliations:** Institute of Pharmaceutical Sciences, Albert-Ludwigs-Universität Freiburg, Hermann-Herder-Straße 9, 79104 Freiburg, Germany

**Keywords:** machine learning, structure and sequence based, druggable cysteine, reactivity prediction

## Abstract

The cysteine side chain has a free thiol group, making it the amino acid residue most often covalently modified by small molecules possessing weakly electrophilic warheads, thereby prolonging on-target residence time and reducing the risk of idiosyncratic drug toxicity. However, not all cysteines are equally reactive or accessible. Hence, to identify targetable cysteines, we propose a novel ensemble stacked machine learning (ML) model to predict hyper-reactive druggable cysteines, named HyperCys. First, the pocket, conservation, structural and energy profiles, and physicochemical properties of (non)covalently bound cysteines were collected from both protein sequences and 3D structures of protein–ligand complexes. Then, we established the HyperCys ensemble stacked model by integrating six different ML models, including K-nearest neighbors, support vector machine, light gradient boost machine, multi-layer perceptron classifier, random forest, and the meta-classifier model logistic regression. Finally, based on the hyper-reactive cysteines’ classification accuracy and other metrics, the results for different feature group combinations were compared. The results show that the accuracy, F1 score, recall score, and ROC AUC values of HyperCys are 0.784, 0.754, 0.742, and 0.824, respectively, after performing 10-fold CV with the best window size. Compared to traditional ML models with only sequenced-based features or only 3D structural features, HyperCys is more accurate at predicting hyper-reactive druggable cysteines. It is anticipated that HyperCys will be an effective tool for discovering new potential reactive cysteines in a wide range of nucleophilic proteins and will provide an important contribution to the design of targeted covalent inhibitors with high potency and selectivity.

## 1. Introduction

The design of targeted covalent inhibitors (TCIs) has received increasing attention in the last decade. Recognizing the advantages of covalent inhibition, such as, on the one hand, greater selectivity and inhibition and, on the other hand, lower dosage and drug resistance, TCIs are increasingly becoming a focal point in the field of kinase-targeted cancer therapies. Nowadays, this approach has been expanded to other therapeutic areas, including autoimmunity, neurology, cardiovascular diseases, gastrointestinal disorders, and inflammation [1,2,3,4,5]. Typical reactive amino acids are serine, threonine, tyrosine, lysine, and, most importantly, cysteine, which is one of the two sulfur-containing amino acids. As the most nucleophilic of the 20 canonical amino acid residues, covalent inhibitors targeting reactive cysteines are becoming increasingly significant in drug discovery and development.

In early cysteine reactivity prediction studies, sequence-based approaches were widely utilized because the structural coverage of proteins possessing reactive cysteines was still largely incomplete, and high-quality databases of covalent 3D protein–ligand complexes were unavailable at that time [6,7]. Advances in technology and proteomics led to the development of quantitative activity-based protein profiling (ABPP), which allows for the high-throughput identification of reactive cysteines in proteins and the quantification of their reactivity [8]. The wealth of such data in the literature provides a benchmark data set for the prediction of cysteine reactivity using sequence-based machine learning (ML) algorithms. In addition, several tools have been developed for functional cysteine prediction, such as DeepCys and Cy-preds [9,10]. However, these methods usually globally characterize the structural cysteines’ (i.e., stable disulfide-bonded cysteines) function in the proteome. Although several computational methods are available to predict functional cysteines, the core problem of finding residues that can be targeted and are suitable for assembling highly reactive warheads is not yet solved. In addition, sequence-based prediction models do not consider the spatial environment’s influence on the reactive cysteines.

As more and more 3D structures of proteins are solved and deposited in the Protein Data Bank (PDB), several studies on the structure-based predictions of targetable cysteine have been reported [11,12]. In 2017, Zhang et al. developed a support vector machine (SVM) model to predict the reactivity of cysteines, for a given protein structure, suitable for TCI design. This analysis pointed out that covalently modified cysteines have unique features compared to cysteines without covalent ligand attachments, including lower acid dissociation constant (pKa), larger solvent-accessible surface area (SASA), and higher frequencies of hydrogen bonding; all of these favor covalent bond formation. Moreover, the authors concluded that the number and type of amino acids surrounding the reactive cysteine affect covalent bond formation.

**Figure 1:**
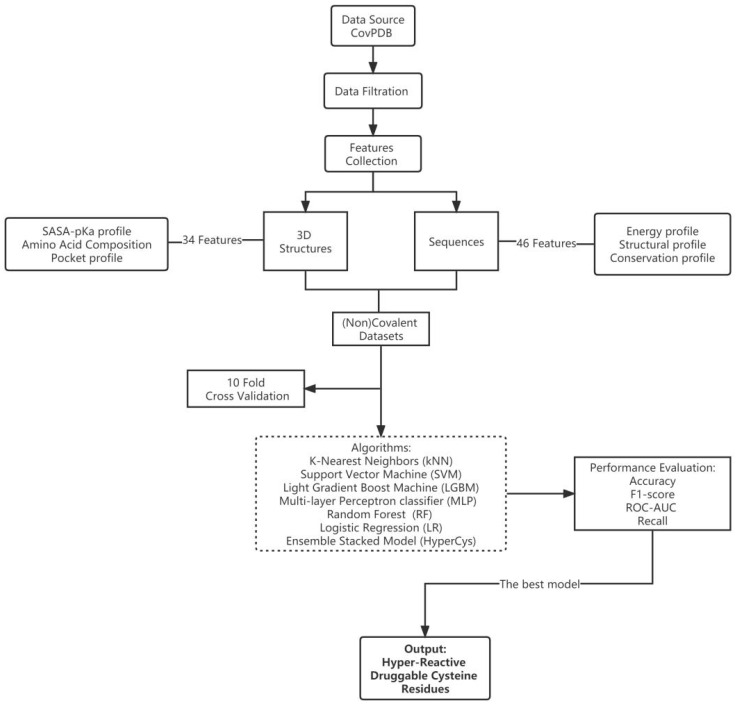
The machine learning workflow of this study.

The sequence-based approach allows for the characterization of cysteine conservation profiles, secondary structural profiles, and site-specific energy profiles [13]. Moreover, the structure-based approach allows for the consideration of environmental effects on reactive cysteines. Both approaches have different functions. Therefore, based on the integration of these two methods, higher-quality benchmark datasets, more relevant features, and a robust method for the prediction of reactive cysteines could be developed. The recently reported CovPDB is the most comprehensive database of covalent protein complexes manually annotated from the Protein Data Bank (PDB) to date, with cumulative information on the individual proteins and ligands [14,15]. The nucleophilic proteins with targetable cysteines in this database were used as a benchmark dataset for developing the reactive cysteine prediction ML algorithm. Protein sequences were downloaded from PDB according to the chain ID in which the covalent cysteines were located, and (non)covalent cysteines in the detected binding pockets were then accurately collected and labelled. Based on a comprehensive analysis of the 3D structures and sequences of the (non)covalent cysteines, we developed a stacked ensemble model for predicting the hyper-reactive cysteines, called HyperCys. We believe this work is an important step in theoretical protocols for hyper-reactive druggable cysteine reactivity prediction and will contribute to the design of TCIs with high potency and selectivity. Figure 1 illustrates the workflow of the proposed method.

## 2. Results and Discussion

### 2.1. Feature Analysis

#### 2.1.1. Feature Statistical Analysis

Statistical analysis (Table 1) shows that the pKa value was 11.18 ± 2.0 pK units for druggable cysteines within proteins and 11.34 ± 1.8 pK units for the cysteines in the NonCovalent dataset, indicating only small differences in reactivity. These covalent cysteines have very high exposure to solvent molecules, elevating the possibility of drug molecules binding to targeting amino acids.

#### 2.1.2. Feature Importance Analysis

The following ML classifiers were used in this study: KNN, SVM, LGBM, MLP, LR, and RF. These classifiers were trained to find the optimum hyperparameters and configurations. The algorithm was performed with Scikit-learn, which provides a GridSearch approach along with 10-fold CV to optimize the hyperparameters for each classifier under consideration. The optimum parameters obtained for each ML model after GridSearch implementation are summarized in Table S1.

**Figure 2:**
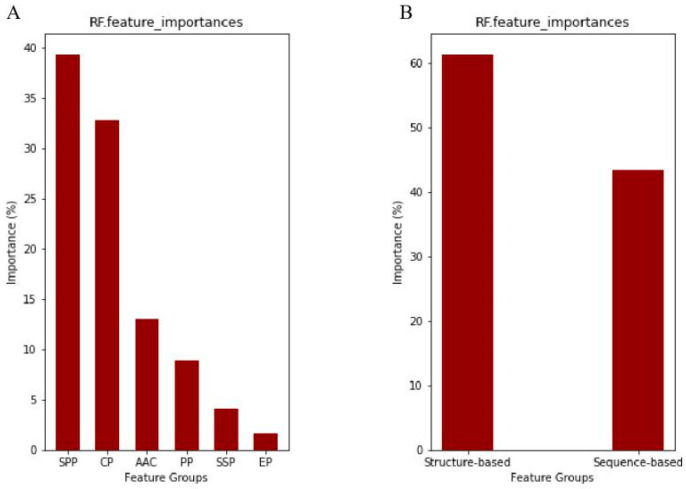
Feature importance for different feature profiles based on the RF classifier: (**A**) for six feature groups and (**B**) for structure-based (SPP + AAC + PP) and sequence-based (CP + SSP + EP), separately.

Feature variables play an important role in creating predictive models, whether a regression or a classification model. Feature importance is a technique that provides a relevant score for every feature variable and can be used to decide which features are least or most important for predicting the target variable. We calculated the feature importance of each feature group using the RF classifier to determine which feature profile contributes the most to our proposed model and to improve model performance. The grouped feature importance of each feature profile utilized in this investigation to predict hyper-reactive cysteines is shown in Figure 2A. The SPP feature group (the SASA and pKa combination) is the most significant feature, followed by the CP, ACC, PP, SSP, and EP groups. On the other hand, Figure 2B shows that structure-based feature groups contribute more to the model than sequence-based ones.

### 2.2. Training ML Classifiers and Generating an Ensemble Stack Model

#### 2.2.1. Different ML Models

We evaluated our model with 10-fold CV. The validation results illustrate the validity and robustness of those models. We developed a stacked model that aggregates the predictions of the base classifiers as a final step after training and assessing models made using the separate ML classifiers. This meta-model was trained using LR. The binary classification ensemble stack model demonstrated an accuracy of 0.758, an F1 score of 0.721, a recall score of 0.692, and a ROC AUC score of 0.818, as shown in Table 2. Regarding accuracy, F1, and ROC AUC, the stacked model (HyperCys) is slightly better than each of the individual models.

#### 2.2.2. Different Window Sizes

The improved PSSM profile used in this paper can incorporate both the evolutionary information and the local environmental information. To optimize window size for predicting hyper-reactive druggable cysteines in a protein, we developed the HyperCys model using different window sizes from 1 to 23. We obtained a maximum accuracy of 0.784, with an ROC AUC of 0.824 for the 21-amino acid window size shown in Table S2, which is the method that achieved the best performance and was selected for the final model (Figure S1).

### 2.3. Application of an Ensemble Stacked Model Generates Accurate Predictions with Different Feature Groups

Table 3 illustrates that gradually incorporating the feature group into the input descriptors could increase HyperCys’ performance. As we gradually expand the feature sets in accordance with the value of the feature importance, the ROC AUC, F1 score, and accuracy all increase for most models, as shown in Table 3. Only the sequence-based feature combination presents considerably lower evaluation indices of 0.556–0.638. The structure-based features are much better than the sequence-based features, which is also consistent with the study of the feature importance of the RF. After incorporating 3D structural features, the accuracy was greatly improved from 0.626 to 0.784 for HyperCys. Table 3 also demonstrates that all feature profiles contribute to the ultimate ROC AUC of 82.6%. It is also shown that the standalone sequence-based machine learning method achieves an accuracy of 0.556 to 0.638. Therefore, pure sequence-based methods are subject to large uncertainties. The combination is more accurate for predicting hyper-reactive cysteine in targets compared with other ML models, which apply only sequenced-based or only 3D structural features.

## 3. Materials and Methods

### 3.1. Data Source

The covalent cysteine dataset (Cov-Set) was collected from the CovPDB database, a manual collection of high-resolution covalent protein–ligand (cP-L) complexes we had previously created [14]. Other cysteines on the same target constitute the noncovalent cysteine dataset (NonCov-Set). The sequences of each target extracted from the PDB, according to the chain IDs and the (non)covalent cysteines in the detected binding pockets, were carefully annotated. In the CovPDB, there are 959 cP-L complexes in which the ligands’ targets are cysteines. In the benchmark dataset, we utilized CD-HIT to cut off those sequences with ≥50% sequence identity [16]. Finally, 304 cysteines were involved in this study, including 135 covalent and 169 noncovalent cysteines for 147 unique cP–L complexes. More information about the dataset statistics is described in Table 4.

### 3.2. Descriptors

In this study, we collected cysteine descriptors at two levels. Firstly, the physico-chemical properties of (non)covalent cysteines were extracted from high-resolution cP–L complexes. Specifically:

(1) Fpocket was used to determine whether or not amino acids are located in detectable binding pockets, and only those cysteines located in pockets were retained [17]. Pocket features, such as druggability, hydrophobicity, and polarity, were collected.
(2) With the help of a PyMOL (Schrödinger LLC, USA) Python script, the total number of surrounding amino acid residues within 4, 6, 8, and 10 Å of cysteine and the respective number of polar and hydrophobic amino acids, according to the hydrophobic/polar (H/P) classification by Kamtekar et al., were calculated [18].
(3) The pKa of each cysteine was calculated because it affects the rate of covalent modification. In theory, cysteines with low pKa values are more vulnerable to covalent alteration [11]. Furthermore, we calculated SASA, a measure of how much of the area of a molecule is available to the solvent. A cysteine with a high SASA value may easily be polarized, increasing the chance for a ligand with a proper warhead to form a covalent bond. The pKa and SASA values for each cysteine and its surrounding amino acids within 4, 6, 8, and 10 Å of the cysteine were calculated using PROPKA 3 and freeSASA, respectively [19,20].
(4) Depth was used to calculate the depth of cysteine burial [21].

**Figure 3:**
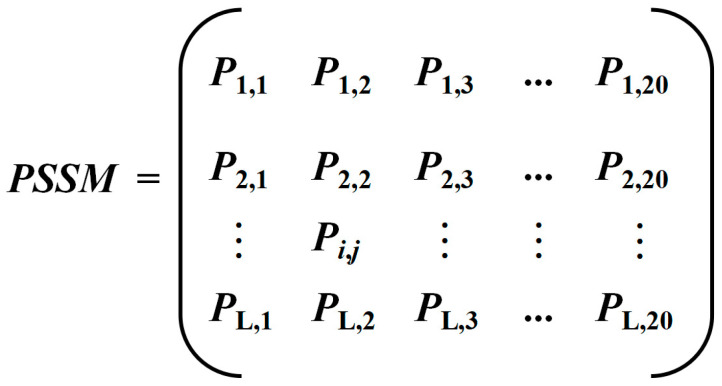
The schematic of a PSSM.

Secondly, in order to create an effective ML model, we extracted various features in cysteine conservation, structural information, and site-specific energy profiles derived from the sequence information. Specifically:

(1) The position-specific scoring matrix (PSSM) corresponding to each protein was mainly generated by the PSI-Blast tool [22]. PSSM-based feature descriptors have successfully been applied to improve the performance of various predictors of protein attributes. The PSSM consists of position-specific conserved scores of amino acids. For each query protein sequence, PSI-BLAST was used to search within the non-redundant NCBI Protein Database (https://ftp.ncbi.nlm.nih.gov/blast/db/, accessed on 15 June 2021) in three iterations with an E-value < 10^−3^ to generate a PSSM. We calculated the L × 20 PSSM for each protein sequence, where L is the length of the sequence. Each residue is represented by a 20-dimensional integer-valued vector shown in Figure 3. The PSSM is generated by counting the frequency of each amino acid observed at each position in multiple sequence motifs, which is usually the log-likelihood ratio of the frequencies of 20 amino acids. According to the principle of additivity, the similarity of any given sequence to a known modality can be measured by calculating the sum of the likelihood scores of the actual occurrence of amino acids at each position.

For an element *P*_i,j_ in PSSM, its value indicates the probability that the amino acid at position i of the sequence mutates to the *j*th amino acid during the evolutionary process. If the value is positive, it indicates a higher probability; otherwise, it indicates a lower probability [23].

(2) The conserved evolutionary information provided by PSSM was expanded to compute one monogram (MG) feature matrix (1 × L) and bigram (BG) feature matrix (20 × L) for each sequence. Those features of monogram-bigram were extracted from the PSSM updated consensus sequence and represented the probability that one amino acid was replaced by another [24].
(3) DisPredict2 v1.0 and SPINE-X v1.0 were used to predict accessible surface area (ASA) and secondary structure (SS) probabilities for helices, coils, and β-folds from sequence information alone [13,25].
(4) The position-specific estimate energy (PSEE) score for each amino acid was calculated using the method described by Iqbal et al. [13]. PSSE scores are generally used to detect the presence of functional binding regions of proteins.

Overall, we collected 36 features from 3D structures of high-resolution cP–L complexes and 46 features from their sequences. Features are summarized in Table 5.

### 3.3. Feature Window Size Selection

**Figure 4:**
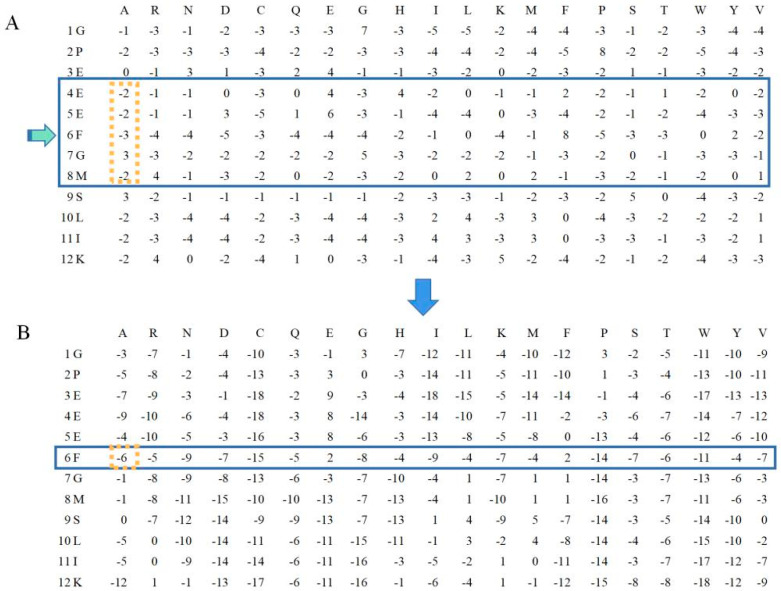
Examples of (**A**) standard PSSM and (**B**) smoothed PSSM.

The structural state of a residue is not solely dictated by the amino acid residue itself but is additionally influenced by nearby residues. We applied a smoothed PSSM encoding scheme with sliding window sizes (ws) from 3 to 23 amino acid lengths with a step of 2 to obtain nearby residue information. The feature vector of a residue αi is represented by the summation of ws surrounding row vectors (Vi − (ws − 1)/2,..., Vi,..., Vi + (ws − 1)/2). For the N terminal and C terminal of a protein, (ws − 1)/2 ZERO vectors, consisting of 20 zero elements, are appended to the hand or tail of a PSSM profile. For example, as shown in Figure 4, when Vi = 6, ws = 5, the smoothed PSSM becomes (−2) + (−2) + (−3) + 3 + (−2) = −6 at the first position, followed by residues calculated in the same way [23].

### 3.4. Machine Learning Methods

We used a stacking-based ML technique to construct HyperCys, which was recently successfully employed to solve diverse bioinformatics challenges [26,27]. Stacking is an integration-based ML technique that integrates inputs from numerous models at various stages to create a new model. It is thought that stacking produces more accurate results than standalone ML approaches because the information collected from numerous prediction models reduces generalization mistakes. Six machine learning models, namely k-nearest neighbors (kNN), logistic regression (LR), support vector machine (SVM), light gradient boost machine (LGBM), multi-layer perceptron classifier (MLP), and random forest (RF), are applied in the suggested system to build the stacking model.

Tuning the hyperparameters of the models is one of the most difficult aspects of constructing ML models. This is because different hyperparameter settings can result in varying levels of accuracy. In order to acquire the best parameter tuning, each learning model was subjected to a GridSearch along with 10-fold cross-validation (CV).

### 3.5. Performance Evaluation

Accuracy refers to the proportion of the number of samples that the model predicts correctly (including TP and TN) to the number of samples in the population, defined as:

(1) $$\frac{\mathrm{TP}\;+\;\mathrm{TN}}{\mathrm{TP}\;+\;\mathrm{FN}\;+\;\mathrm{TN}\;+\;\mathrm{FP}}$$

The F1 score is a statistical measure of the accuracy of binary classification (or multitask dichotomous) models. It takes into account both the accuracy and recall of the classification model, defined as:

(2) $$\frac{\mathrm{TP}\;}{\mathrm{TP}\;+\;\mathrm{FN}\;}$$

The receiver operating characteristic area under the curve (ROC AUC) measures the area beneath the ROC curve. The ability to appropriately identify random positive and negative data was measured in this domain.

## 4. Conclusions

Herein, we report the development of the first ensemble stacked model (HyperCys) for predicting hyper-reactive druggable cysteines based on both protein 3D structures and sequences. This method is powerful, as it blends a heterogeneous group of algorithms to expose distinct yet complementary aspects of the data. This study used ML algorithms to determine the optimal combination of protein features. The ensemble algorithm is based on six algorithms that have previously been shown to be effective classification algorithms. It is anticipated that HyperCys will be an effective tool for discovering new potential reactive cysteines for a wide range of targeted proteins.

## Figures and Tables

**Table 1:** Statistical analysis of pKa and SASA of cysteines in NonCov-Set and Cov-Set datasets.

Feature	Groups	Min	Q1	Median	Q3	Max	Mean	SD
SASA	NonCov-	0.09	5.54	12.15	24.45	112.51	19.01	21.61
	Set							
SASA	Cov-Set	4.18	15.359	21.53	37.415	122.09	28.62	20.73
pKa	NonCov-	5	10.32	11.19	12.13	19.76	11.34	1.8
	Set							
pKa	Cov-Set	6.79	9.99	10.88	12.02	20.01	11.18	2.0

**Table 2:** Performance of six ML models for the prediction of hyper-reactive druggable cysteines involving all feature groups after 10-fold CV (ws = 1).

Model	ACC	F1	RECALL	ROC AUC
KNN	0.685	0.613	0.571	0.771
LR	0.740	0.704	0.701	0.812
SVM	0.734	0.701	0.704	0.804
LGBM	0.757	0.711	0.691	0.821
RF	0.738	0.685	0.677	0.809
MLP	0.756	0.723	0.676	0.845
HyperCys	0.758	0.721	0.692	0.818

**Table 3:** ML models’ accuracy performance for different feature combinations with the best window size (ws = 21).

Feature Combination	Metric	KNN	LR	SVM	LGBM	RF	MLP	HyperCys
sequence-based	ACC	0.608	0.632	0.638	0.565	0.592	0.556	0.626
	F1	0.521	0.563	0.520	0.486	0.493	0.014	0.496
	RECALL	0.482	0.534	0.445	0.468	0.488	0.008	0.385
	ROC AUC	0.624	0.661	0.674	0.583	0.574	0.656	0.670
structure-based	ACC	0.684	0.707	0.721	0.760	0.743	0.694	0.757
	F1	0.577	0.637	0.669	0.718	0.681	0.603	0.714
	RECALL	0.496	0.589	0.654	0.698	0.674	0.542	0.681
	ROC AUC	0.718	0.783	0.765	0.836	0.819	0.781	0.823
structure- & sequence-based	ACC	0.704	0.747	0.710	0.770	0.741	0.783	0.784
	F1	0.633	0.704	0.669	0.722	0.674	0.727	0.754
	RECALL	0.570	0.680	0.669	0.683	0.653	0.743	0.742
	ROC AUC	0.777	0.801	0.799	0.847	0.814	0.852	0.824

**Table 4:** Overview of the dataset statistics.

Attributes	Count
cP–L complexes	147
Different covalent mechanisms	14
Pre-reactive ligands	134
Warheads	40
Ligand types	6
Nucleophilic proteins	147
Protein classes	9
Collected features	80
Covalent cysteines	135
Noncovalent cysteines	169

**Table 5:** Feature description.

Feature Type	Feature Group	Size	Feature
Structure-based	SASA-PKA Profile (SPP)	18	10Å.pka.total; 10Å.pka.ave;
			10Å.SASA.total;
			10Å.SASA.ave; 8Å.pka.total;
			8Å.pka.ave; 8Å.SASA.total;
			8Å.SASA.ave; 6Å.pka.total;
			6Å.pka.ave; 6Å.SASA.total;
			6Å.SASA.ave; 4Å.pka.total;
			4Å.pka.ave; 4Å.SASA.total;
			4Å.SASA.ave;
			10Å.pka.total; 10Å.pka.ave;
			10Å.SASA.total;
			10Å.SASA.ave; 8Å.pka.total;
			8Å.pka.ave; 8Å.SASA.total;
			8Å.SASA.ave; 6Å.pka.total;
			6Å.pka.ave; 6Å.SASA.total;
			6Å.SASA.ave; 4Å.pka.total;
			4Å.pka.ave; 4Å.SASA.total;
			4Å.SASA.ave; SASA; pKa
Structure-based	Pocket Profile (PP)	4	Atom Depth; Drug Score;
			Hydrophobicity Score;
			Polarity Score
Structure-based	Amino Acid Composition (AAC)	12	10Å.Total; 10Å.H; 10Å.P;
			8Å.Total; 8Å.H; 8Å.P; 6Å.Total;
			6Å.H; 6Å.P; 4Å.Total; 4Å.H;
			4Å.P
Sequence-based	Conservation Profile (CP)	41	20 PSSMs; monograms; 20
			bigrams
Sequence-based	Energy Profile (EP)	1	PSEE
Sequence-based	Secondary Structural Profile (SSP)	4	Helix probability; Beta-Strand
			probability; Coil probability;
			ASA

## Data Availability

Data are openly available in a public repository. The data that support the findings of this study are openly available at https://github.com/mingjie-tech/HyperCys_v1.0 (accessed on 1 January 2023).

## Supplementary Materials

The following supporting information can be downloaded at: https://www.mdpi.com/article/10.3390/ijms24065960/s1.
