# Supplementary material for: HyperCys: A Structure- and Sequence-Based Predictor of Hyper-Reactive Druggable Cysteines
Source: Int J Mol Sci. 2023 Mar 22;24(6):5960. doi: 10.3390/ijms24065960 (PMC10054327; doi:10.3390/ijms24065960)
Supplement: Supplementary file 1 [file ijms-24-05960-s001.zip › ijms-2249908-supplementary.pdf]

Table S1. The hyperparameter values of the ML models.

Model	Hyperparameters	Search Domain	Final Setting
KNN	n_neighbors	Range (1, 100)	9
SVM	C	[0.1, 1, 10, 20, 100, 1000]	100
	kernel	[‘rbf’, ‘poly’, ‘sigmoid’]	sigmoid
	gamma	[1, 0.1, 0.01, 0.001, 0.0001]	0.001
LGBM	n_estimators	[100, 200, 300, 400, 700, 1000]	100
	colsample_bytree	[0.1, 0.3, 0.5, 0.7, 0.8]	0.3
	max_depth	[15, 20, 25, 50, 70, 90]	15
	num_leaves	[50, 100, 200]	50
MLP	hidden_layer_sizes	[(50,50,50), (50,100,50)]	(50, 50, 50)
	solver	[’sgd’, ’adam’]	adam
	alpha	[0.00001,0.0001,10,100,1000]	10
	learning_rate	[’constant’,’adaptive’]	adaptive
RF	n_estimators	[10, 80, 100]	80
	criterion	[’gini’, ’entropy’]	gini
	max_depth	[10,500,1000,3000]	3000
	min_samples_split	[2, 3, 4]	4
LR	C	[0.001, 0.01, 0.1, 1, 10, 100]	0.1

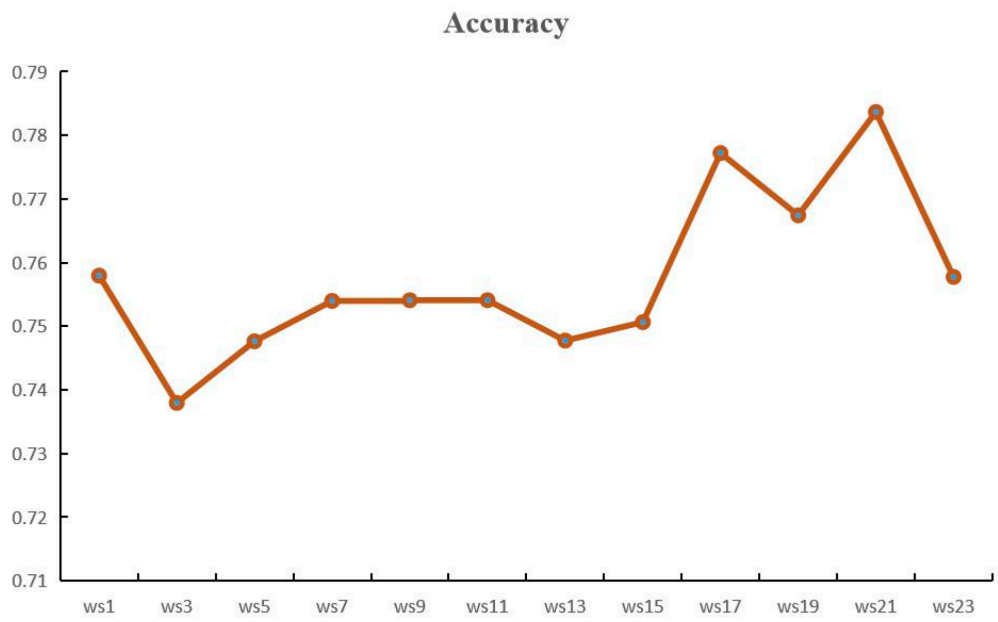

Figure S1. Performance comparisons of the HyperCys model trained using feature vectors created by applying a sliding window of different sizes. The accuracies of the model for various window sizes are reported.

Table S2. Performance of the HyperCys model with different window sizes.

Model	ACC	F1	RECALL	ROC AUC
ws1	0.758	0.721	0.692	0.818
ws3	0.738	0.713	0.647	0.798
ws5	0.748	0.695	0.654	0.805
ws7	0.754	0.713	0.675	0.812
ws9	0.754	0.715	0.661	0.809
ws11	0.754	0.721	0.675	0.805
ws13	0.748	0.701	0.631	0.806
ws15	0.751	0.709	0.675	0.812
ws17	0.777	0.741	0.721	0.807
ws19	0.767	0.732	0.699	0.824
ws21 (the best)	0.784	0.754	0.742	0.824

ws23	0.758	0.726	0.698	0.812
